# Mats Lekander, *The Inflamed Feeling: The Brain's Role in Immune Defense*

**DOI:** 10.1093/emph/eoad022

**Published:** 2023-08-01

**Authors:** Eric C Shattuck

**Affiliations:** Department of Anthropology, Florida State University, Carraway Building, 909 Antarctic Way, Tallahassee, FL 32304, USA; Institute for Health Disparities Research, University of Texas at San Antonio, One UTSA Circle, San Antonio, TX 78249, USA

*The Inflamed Feeling* is a brief tour through psychoneuroimmunology (PNI), a relatively young and interdisciplinary field concerned with understanding the reciprocal links between the brain, psychological processes, and the immune system. PNI is particularly ripe for evolutionary approaches (particularly given the selective pressure that infectious diseases are thought to have on human evolution), although most research tends toward outlining mechanisms and specifying the biological connections between the systems and processes under its purview. By stepping back to consider how the brain and immune system interact in a variety of real-world contexts, this book is a step toward applying explicit evolutionary thinking to PNI. The book is broadly divided into three parts: the brain-body connection (chapters 1 and 2), bio-behavioral immunity/defense (chapters 3, 4, and 5), and psychosomatic health, broadly construed (chapters 6 to 10).

The first section explores the role of prediction and error in the brain/mind (what has been termed the ‘Bayesian brain’, or the idea that the brain maintains internal probabilistic models of the world that are frequently updated based on new, incoming information) through examples of visual illusions, some of which (e.g. the ‘Cheshire Cat’ illusion) could be an effective in-class teaching demonstration. Lekander then applies this Bayesian thinking to connections between the brain and body with a particular eye for how this might influence health. He sums up this central argument—that perception and context play a key role in health—nicely: ‘The body is a hypothesis—once again, one that is normally reasonable and well-functioning, but sometimes completely inaccurate, with a potential for both ill health and recovery that is of great relevance to medical practice (pg. 33)’. The application of Bayesian logic, currently in vogue in psychology, to immune function is a fascinating avenue for generating new research questions and a key contribution of Lekander’s book.

In keeping with the conversational tone of the book, Chapter 3 provides a broad, high-level introduction to the immune system. Although it would be easy to get bogged down in cell types, signaling molecules, and the other mechanisms of PNI, Lekander manages to provide sufficient context for readers not versed in immunology to follow along. Similarly, the chapter includes an introduction on sleep, stress, and immunity that outlines the biological and (although not explicitly stated) evolutionary logic of the relationship between physical and psychosocial stress and changing immune function. While this chapter is generally effective, providing additional citations to introductory immunology texts for interested readers would have been a welcome addition. Including some of the considerable literature on stress and immune function in non-human animals would also have demonstrated the wide applicability of PNI. Chapter 4 covers the ‘sickness response’, or sickness behavior—a constellation of behavioral changes occurring during inflammatory states. This chapter covers the classical thinking regarding sickness behavior: that it serves to reduce energy expenditure on temporarily unnecessary social behaviors, minimize exposure to predators in a weakened state, and so on. However, additional research has shown that sickness behavior is responsive to social conditions and competing adaptive drives, albeit predominantly in non-human animal models (e.g. [1], among others). Including a discussion of this work would have provided additional support for the contextual nature of health and sickness, Lekander’s key argument.

The last chapter of the bio-behavioral immunity section covers the role of disgust and prejudice in a ‘total defense system against infection (p. 83)’, that is the ‘behavioral immune system’ [2]. It is probably the most evolutionarily engaged chapter in the book and covers many of the ‘popular highlights’ of behavioral immune system research, such as connections between prejudice, discrimination, and disease avoidance. However, it was surprising not to see research indicating that outward cues of disease could activate both the behavioral and the ‘classic’ immune system, for example that exposure to disease stimuli can elicit a stronger stimulated inflammatory response [3]. Nevertheless, the inclusion of the behavioral immune system in a book dedicated to discussing biological immunity is a welcome contribution and illustrates the advancements in thinking about immunology based on PNI and allied, interdisciplinary efforts.

The final—and longest—section deals broadly with psychosomatic health, ranging from self-rated health, inflammation, and interoception (i.e. the sense of the internal body; Ch. 6) to social and cultural influences on health and wellbeing (Ch. 9). As this is a slightly more speculative portion of the book, it is these chapters that readers are likely to find helpful in generating novel research questions. For instance, Chapter 7 suggests that ‘feeling sick’, or the sickness response, could be akin to an emotion. Lekander draws heavily from LeDoux’s view [4] that emotions are adaptive reactions to survival threats. In this view, the emotional aspect of feeling sick is one part of a ‘single integrated defense network [that] has emerged through gradual evolutionary adaptation (p. 143)’ and is therefore likely inextricable from the biological immune system, the keystone of that network. Schrock *et al*. [5] published something similar, arguing that lassitude, or ‘feeling sick’, could be considered an emotion from an evolutionary psychology perspective. While this paper is not cited or discussed in this chapter, it is telling that separate researchers have arrived at roughly the same conclusions. As Lekander notes, approaching sickness/lassitude through the lens of emotion can advance our understanding of ill health through understanding how the sickness response can be modulated by ‘context, interpretation, and other factors (p. 151)’. This functional approach to sickness is an area where evolutionary medicine, evolutionary psychology, and anthropology can make significant contributions.

In all, *The Inflamed Feeling* is an approachable, conversationally written book that neatly encapsulates the intersection of inflammation, immunity, and behavior. This, combined with its relatively short length and low price, make it a good option for undergraduate classes or early graduate seminars on psychoneuroimmunology or health psychology. Similarly, researchers new to PNI may also find it useful as an introduction and inspiration for their own research questions, though it would need to be supplemented by more in-depth texts.
